# The Diplodia Tip Blight Pathogen *Sphaeropsis sapinea* Is the Most Common Fungus in Scots Pines’ Mycobiome, Irrespective of Health Status—A Case Study from Germany

**DOI:** 10.3390/jof7080607

**Published:** 2021-07-27

**Authors:** Kathrin Blumenstein, Johanna Bußkamp, Gitta Jutta Langer, Ewald Johannes Langer, Eeva Terhonen

**Affiliations:** 1 Forest Pathology Research Group, Department of Forest Botany and Tree Physiology, Faculty of Forest Sciences and Forest Ecology, Büsgenweg 2, Georg-August-University Göttingen, 37077 Göttingen, Germany; kathrin.blumenstein@uni-goettingen.de; 2 Section Mycology and Complex Diseases, Department of Forest Protection, Northwest German Forest Research Institute, Grätzelstr. 2, 37079 Göttingen, Germany; johanna.busskamp@nw-fva.de (J.B.); gitta.langer@nw-fva.de (G.J.L.); 3 Department of Ecology, Institute for Biology, Faculty of Mathematics and Sciences, Universität Kassel, Heinrich-Plett-Straße 40, 34132 Kassel, Germany; elanger@uni-kassel.de

**Keywords:** endophytes, mycobiome, MiSeq Illumina mycobiome, *Pinus sylvestris*, high-throughput sequencing (HTS), culture-based endophytic community

## Abstract

The opportunistic pathogen *Sphaeropsis sapinea* (≡*Diplodia sapinea*) is one of the most severe pathogens in Scots pine, causing the disease Diplodia tip blight on coniferous tree species. Disease symptoms become visible when trees are weakened by stress. *Sphaeropsis sapinea* has an endophytic mode in its lifecycle, making it difficult to detect before disease outbreaks. This study aims to record how *S. sapinea* accumulates in trees of different health status and, simultaneously, monitor seasonal and age-related fluctuations in the mycobiome. We compared the mycobiome of healthy and diseased Scots pines. Twigs were sampled in June and September 2018, and filamentous fungi were isolated. The mycobiome was analyzed by high-throughput sequencing (HTS) of the ITS2 region. A PERMANOVA analysis confirmed that the mycobiome community composition significantly differed between growth years (*p* < 0.001) and sampling time (*p* < 0.001) but not between healthy and diseased trees. *Sphaeropsis sapinea* was the most common endophyte isolated and the second most common in the HTS data. The fungus was highly abundant in symptomless (healthy) trees, presenting in its endophytic mode. Our results highlight the ability of *S. sapinea* to accumulate unnoticed as an endophyte in healthy trees before the disease breaks out, representing a sudden threat to Scots pines in the future, especially with increasing drought conditions experienced by pines.

## 1. Introduction

The impacts of forest pathogens and pests are increasing dramatically worldwide. Changes in the climate can lead to the development of optimal conditions for fungal pathogens and hence to severe declines in native and non-native tree species [1]. *Sphaeropsis sapinea* (Fr.) Dyko & Sutton, Botryosphaeriaceae, Botryosphaeriales Theiss. & Syd. (most common synonym: *Diplodia sapinea* (Fr.) Fuckel) is the causal agent of Diplodia tip blight (Sphaeropsis tip blight) in conifers. The anamorphic fungus was first described as *Sphaeria sapinea* Fr., collected in Sweden from *Abies* sp. and *Pinus* sp. by Fries [2]. The correct name of this anamorphic Botryosphaeriaceae is under discussion [3,4], but the current name after Index Fungorum is still *S. sapinea*.

Within its life cycle, *S. sapinea* has different trophic stages [5]. It can live asymptomatically as an endophyte in its host tree [6,7], and can transform from a latent to an opportunistic pathogen [8,9] or/and saprotroph [10]. In combination with stress-inducing factors, such as drought, hail, extreme temperatures, or mechanical wounding [11,12], *S. sapinea* may rapidly become pathogenic, leading to sudden disease outbreaks [13,14,15]. Ghelardini et al. [16] considered that cryptic and latent pathogens, such as *S. sapinea*, are the most important drivers of emerging fungal diseases in forests. *Sphaeropsis sapinea* is a threat in the Northern Hemisphere, as indicated by several reports of new outbreaks in North America, e.g., [14,17,18]; Central Europe [15,19,20]; and Southern Europe [21,22]. Recently, a sudden disease outbreak and invasion of Northern Europe was observed in Estonia by Hanso and Drekhan [23] and in Sweden by Oliva et al. [24] and Brodde et al. [25]. In Finland, *S. sapinea* has been found as a saprotroph on cones [26] and as an asymptomatic endophyte in shoots of healthy Scots pine [7]. The growth of *S. sapinea* is favored by a warmer climate [8], and it becomes more aggressive when the host is under drought stress [13]. Because of the endophytic stage of *S. sapinea,* the accumulation of this pathogen can proceed unnoticed before disease outbreaks [25]. It is noteworthy that the origin of *S. sapinea* remains unknown, although it was most likely introduced to new regions with the movement of seeds or symptomless host material [14,17,27].

**Figure 1:**
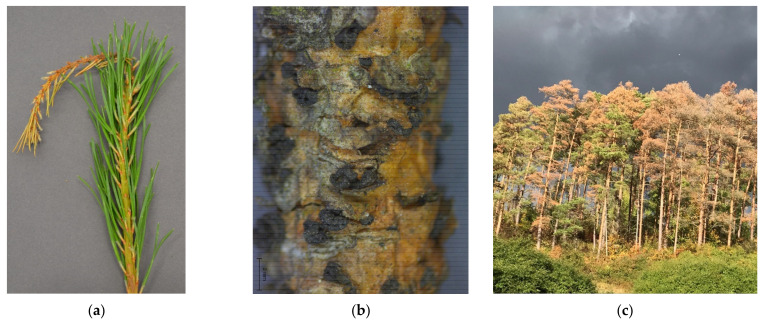
*Sphaeropsis sapinea*—Diplodia tip blight symptoms: (**a**) diseased Scots pine twig with dieback of the current shoot; (**b**) after seedling blight, black pycnidia can be observed on the twigs; (**c**) affected Scots pine stand in Germany.

Several conifer species, particularly members of the genus *Pinus*, are the main hosts [28] of *S. sapinea*. Over 33 *Pinus* spp. are known to be susceptible [10]. In Europe, native pine species such as Austrian pine (*Pinus nigra* J.F. Arnold), Mountain pine (*Pinus mugo* Turra), and Scots pine (*Pinus sylvestris* L.) are most susceptible [19,29,30]. Recently, *S. sapinea* has also been found as an endophyte in broadleaved trees such as *Fagus sylvatica* [31,32]. As local environments are changing, Scots pine trees in Germany have to face the loss of vitality due to expected increasing frequency of droughts, exacerbated by long periods of high temperatures and intense solar radiation [13]. Scots pines stressed by drought and/or hail have been found to be especially sensitive to disease outbreaks [9,13,33]. Typical disease symptoms of Diplodia tip blight are brown and short-needled, dead current-year shoots (Figure 1a) [34], resinous cankers on main stems and branches (branch and bole canker), dieback, and misshapen tops. *Sphaeropsis sapinea* (Figure 1b) can also cause death of cones, seedling blight and sapwood staining [35], damping off and collar rot of seedlings, and root diseases [10], all of which may lead to the death of the entire tree (Figure 1c).

It is commonly acknowledged that trees do not grow alone—they are accompanied by a diverse and as yet unexplored mycobiome, which may have an impact on the health of the hosts [36,37]. The hidden diversity of the mycobiome, defined as all fungal organisms that live inside (endophytes) and across (epiphytes) the host tree’s tissue, may have an extremely important function—the diverse composition of the undiscovered fungi may enhance the fitness of not only individual trees, but also of the whole forest ecosystem [36,38]. In that sense, it is possible that differences in the mycobiota of healthy and sick trees may influence the processes leading to disease outbreak and different disease symptoms [39]. It is therefore important to monitor the ecological and evolutionary dynamics of the host tree–fungal interactions and the influence of *S. sapinea* emergence on the outcome of these interactions [40]. Many studies support the hypothesis that fungal endophytes may enhance the tolerance of the host tree to fungal pathogens [41,42,43,44,45,46,47,48,49,50,51]. In addition to endophytic bacteria and filamentous fungi, yeasts are a unique subset of the symbiotic microbiota within plants [52]. Yeasts may represent a significant component of the mycobiome of Scots pine twigs in temperate regions compared to endophytes, epiphytes (phylloplane and bark community) or saprotrophs [53]. Endophytic yeasts may contribute to the increased growth and health of the host tree by, for example, producing plant hormones [52]. To date, there is only limited information, but yeasts may fulfill different functions in the decomposition of plant materials. All these findings are extremely important, as in the future, there may be an opportunity to use beneficial endophytes (yeasts, filamentous fungi, bacteria) that can act as biocontrol agents against pathogens and, in this case, against *S. sapinea*. Indeed, endophytes are increasingly being considered or exploited as a component of integrated pest management (IPM).

The aim of this study was to monitor *S. sapinea* and the mycobiome of Scots pine twigs of varying health status, collected from a diseased German forest stand. First, we aimed to monitor the accumulation of *S. sapinea* in these trees. Second, we aimed to examine differences in the composition of the fungal communities of symptomless (healthy) and diseased trees (increasing number of symptoms), with a view to identifying the presence of antagonist endophytes in healthy trees. As communities of fungi have been observed to change seasonally and with tissue age [54], we sampled twice during the 2018 growing season (2017 and 2018 growth was collected). Previous isolation studies on fungal communities have recorded 103 outgrowing endophytic fungal species from Scots pine twigs [20]. HTS methods are currently used to determine the fungal assemblages in plant hosts [55,56,57,58]. Therefore, we determined the mycobiome of Scots pine using two different approaches: HTS of the internal transcribed spacer 2 (ITS2) region by MiSeq Illumina sequencing and a culture-based isolation method. The plant material chosen (to compare the mycobiome) comprised asymptomatic Scot pine twigs. After infection, *S. sapinea* grows to the peridermis and cortex (and finally into the vascular tissues in diseased plants), and therefore it can be assumed that the fungus accumulates in the twigs [59].

## 2. Materials and Methods

### 2.1. Sampling Site

Sampling took place in June and September 2018 in a stand of Scots pine (defined as diseased due to *S. sapinea*) (52.327653° N 11.189848° E) close to Behnsdorf in Saxony-Anhalt, Germany. The stand consisted of a pure monoculture of single-layer mature (40-year-old) Scots pine. The area is located 150 m above sea level in a flat position in the Northwestern Harz foreland and in a moderately dry climate zone (9 °C annual mean temperature and 550 mm annual mean precipitation, database: Deutscher Wetterdienst, time period 1981–2010, 2018: 10.58 °C mean temperature and 511 mm mean precipitation and 2017: 9.8 °C mean temperature and 570 mm annual mean precipitation). The substrate is glazed loose rock over rhyolite. The soil in Behnsdorf is very acidic and low in bases, has an intermediate nutrient supply, and has an adequate fresh water supply. The soil type was classified as brown earth and the soil texture “sand silt”. In preliminary studies, the disease intensity in this stand was assessed and divided into six classes (defoliation by percentage 0: 0–5%, 1: >5–20%, 2: 21–40%, 3: 41–60%, 4: 61–80%, 5: 81–99%). In June 2018, 30 representative trees (five trees per disease class) were chosen for sampling (Table 1). By September 2018, eight trees sampled in June were dead, and an attempt was made to replace them with new trees in the respective disease class. However, three trees could not be replaced, and thus only 27 trees were sampled in September. Altogether we collected twig samples from 35 different trees, which were cut from a stem height of 6–8 m (114 twig samples, Table 1).

Three randomly selected 2-year-old twigs (growth years 2017 and 2018) per tree were collected. Two twigs were stored in 8 °C and analyzed within 48 h using a culture-based method. One twig per tree was immediately placed in liquid nitrogen at the site and stored at −80 °C before DNA extraction and metabarcoding. On each sampling occasion (June or September), samples were collected for two growth years, 2017 and 2018, from each disease class (Table 1), totaling 114 twig samples for HTS study and 228 for cultivation study.

### 2.2. Culture-Based Isolation, Morphological and Molecular Identification

The twigs were divided into two groups on the basis of growth years, 2017 and 2018. The shoots were defoliated, washed, and surface sterilized as described in Bußkamp et al. [20]. Thereafter, shoots were cut into 5 mm pieces and plated on malt yeast peptone agar (MYP) modified after Langer [60]. The Petri dishes were incubated for up to three weeks at room temperature (at around 22 °C) under a natural day/night cycle. They were visually checked for developing colonies on a weekly basis. Emerging mycelia were sub-cultured separately on MYP. Isolated strains were assigned to mycelial morphotypes and identified on the basis of micromorphological characters. For identifying fungi, a ZEISS Axiostar plus microscope (Zeiss, Omnilab-laborzentrum GmbH & Co.KG, Gehrden, Germany) was used, and standard procedures for fungi described in [61] were followed. In addition to standard literature recommended by Oertel [62] for determination of fungi and forest diseases, other literature was consulted, including [63,64,65,66,67,68,69,70,71,72]. One representative strain of each morphotype was used for molecular identification.

Fungal DNA was extracted for molecular identification following the protocol described by Keriö et al. [73]. Taq DNA polymerase (Fisher Scientific GmbH, Schwerte, Germany) was used for PCR amplification of ITS regions with primer pairs ITS1-F [74] and ITS4 [75]. Briefly, the PCR protocol was as follows: 1X PCR Buffer, 200 µM dNTP, 0.5 µM primer 1, 0.5 µM primer 2, 100 ng template DNA, 0.2 U/µL DNA polymerase; the reaction mixture was adjusted to 25 µL with autoclaved MQ H_2_O. The PCR conditions used for the ITS region were 94 °C for 3 min; 30 cycles of 94 °C for 30 s, 55 °C for 1 min, 72 °C for 1 min, and 72 °C for 10 min. Possible contaminants were determined with a negative control using sterile water as a template in the PCR protocols. StainIN™ RED Nucleic Acid Stain (highQu GmbH, Kraichtal, Germany) was used to confirm DNA amplicons on a 1.5% agarose gel, and the visual assessment was made using ultraviolet transillumination. PCR products were purified and sequenced using the ITS4 primer at Microsynth SEQLAB (Göttingen, Germany). The ITS sequences were extracted with open-source software (https://microbiology.se/software/itsx/, accessed on 15 January 2020) for the ITS2 sub-region from the fungal nuclear ITS sequences [76]. The ITS1 and ITS2 sequences were used for BLASTN [77] searches against GenBank/NCBI [78] to provide taxonomic identification. Intraspecific ITS similarity for the sequenced fungi of 98–100% was used at species level and further confirmed the morphological identification.

### 2.3. Fungal Metabarcoding and Data Analysis

The frozen twigs were divided into two groups on the basis of growth years, 2017 and 2018. The twigs were defoliated, and each sample was ground using a Mixer Mill MM 400 (Retsch GmbH) with a set program of 25.0 Hz for 20 s to prevent thawing of the samples. The samples and the milling equipment were handled with liquid nitrogen throughout the entire milling process. The ground product was then stored in 1.5-mL tubes at −80 °C. DNA was extracted from 50 mg of the homogenized wood sample using an “innuPREP Plant DNA Kit” (Analytik Jena AG, Jena, Germany), according to the manufacturer’s instructions. DNA products were sent to Microsynth SEQLAB (Switzerland). Illumina MiSeq sequencing of amplicons was successful for 95 samples (83%) (Table 1). To sequence the internal transcribed spacer (ITS2) regions of the fungal 18S rRNA gene, we created two-step Nextera PCR libraries [79] using the primer pair ITS3 (5′- GCA TCG ATG AAG AAC GCA GC -3′) and ITS4 (5′- TCC TCC GCT TAT TGA TAT GC -3′) [80]. Subsequently, the Illumina MiSeq platform and a v2 500-cycle kit were used to sequence the PCR libraries. The resulting paired-end reads that passed Illumina’s chastity filter were subject to de-multiplexing and trimming of Illumina adaptor residuals using Illumina’s real-time analysis software included in the MiSeq reporter software v2.6 (no further refinement or selection was undertaken). The quality of the reads was checked with the software FastQC version 0.11.8 [80]. The locus-specific ITS2 primers were trimmed from the sequencing reads with the software cutadapt v2.8 [81]. Paired-end reads were discarded if the primer could not be trimmed. Trimmed forward and reverse reads of each paired-end read were merged to re-form the sequenced molecule in silico, on the basis of a minimum overlap of 15 bases, using the software USEARCH version 11.0.667. Merged sequences were then quality filtered, allowing for a maximum of one expected error per merged read and discarding those containing ambiguous bases. From the remaining reads, the ITS2 subregions were extracted with the help of the ITSx software suite v1.1.2 [76] and its fungi database. The extracted sequences were then denoised using the UNOISE algorithm implemented in USEARCH to form operational taxonomic units (OTUs), discarding singletons and chimeras in the process. The resulting OTU abundance table was filtered for possible bleed-in contamination using the UNCROSS algorithm. OTUs were compared against the reference sequences of the UNITE database, and taxonomies were predicted on the basis of a minimum confidence threshold of 0.5 using the SINTAX algorithm implemented in USEARCH. Rarefaction analysis was performed with the R software modules phyloseq v1.26.1 and vegan v2.5-5. The alpha diversity mean values were employed to create a sample depth-based rarefaction curve (Figure S1). DESeq2 was applied for the normalization of the data [82]. Libraries, sequencing, and data analysis described in this section were performed by Microsynth AG (Balgach, Switzerland). Additional BLAST searches against NCBI GenBank were performed manually.

The normalized HTS data were used for the statistical analysis. For isolates, the exact number of isolates was used. All data analyses were conducted in R version 3.5.1 [83]. For each HTS sample (95) and 114 cultured samples, we calculated the Shannon–Wiener index [84] and the Simpson index [85]. Permutational Multivariate Analysis of Variance (PERMANOVA) in VEGAN version 2.4 [86] was used to investigate the statistical differences/similarities in community structure between HTS and cultured samples (factors: growth year, disease class, sampling time). A permutation test (permutest.betadisper, method = bray) was used to reveal the differences/similarities in dispersion between OTU composition in HTS and isolate data (growth year, disease class, sampling time) in VEGAN version 2.4 [86]. One-way ANOVA was used to investigate the statistical differences/similarities in diversity indices. *S. sapinea* reads were analyzed with PERMANOVA and further with the Kruskall–Wallis test (the Tukey HSD test was used to examine differences between groups) for HTS data. One-way ANOVA was used for isolate data (normally distributed), and the Tukey HSD test was used to examine the differences between groups (in disease classes). A Welch two-sample *t*-test was used for *S. sapinea* isolate data to investigate differences between groups with respect to growth year and sampling time. The statistically different OTUs (factors: growth year, disease class, sampling time) were identified with the R package module “indicspecies” [87]. If more than 10 OTUs were found to be indicator species when disease class was variable, they were used for additional PERMANOVA analysis. The permutation test based on the Bray method was used to visualize their abundance in different disease classes.

The FUNGuild database v1.0 database (https://github.com/UMNFuN/FUNGuild, accessed on 3 February 2020) was used to assess the ecological and functional status of OTUs identified to the species level [88]. Trophic statuses included pathogens (in FUNGuild referred to as pathotrophic fungi), saprotrophs, and mutualists (in FUNGuild referred to as symbiotrophic fungi). However, all fungal taxa were also categorized into trophic levels manually on the basis of the authors’ expertise and a literature study. During manual curation, the following trophic status classifications were used: endophytes, epiphytes, plant pathogens, and wood-decay fungi. The plant pathogen composition for the different disease classes was analyzed using PERMANOVA followed by a permutation test.

## 3. Results

### 3.1. Fungal Isolates Retrieved by the Culture-Based versus the HTS Method

**Figure 2:**
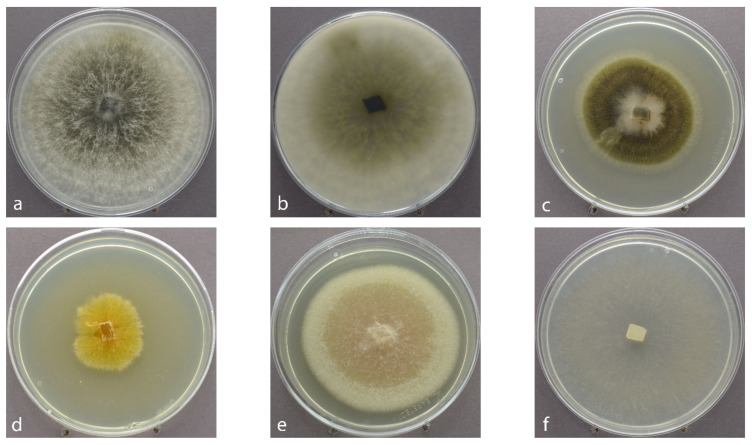
Most frequently isolated endophytes of Scots pine twigs, cultivated on MYP in 90 mm Petri dishes, seven days in ambient daylight at room temperature, at around 22 °C: (**a**) *Sphaeropsis sapinea* obvers; (**b**) *Sphaeropsis sapinea* revers; (**c**) *Sydowia polyspora*; (**d**) *Truncatella conorum-piceae*; (**e**) *Microsphaeropsis olivacea*; (**f**) *Desmazierella acicola*.

From the 228 twigs (including shoots from the years 2017 and 2018, collected from 35 trees) chosen for the isolation study, 1358 segments (June: 740, September: 618) were plated, resulting in 1425 outgrowing fungi. Due to the difference in length between the shoots from 2017 and 2018, various numbers of segments (3–9) per shoot were studied. The mean number of isolated strains of a single shoot varied between 4.95 and 8.3 over all disease classes and both sampling times (Table 1). The mean number of isolated taxa varied between 3.4 and 5.7 species per tree (Table 1). Besides yeasts, which were ignored (1.61% of outgrowing fungi); unidentified ascomycetes (1.4% of outgrowing fungi); and *Penicillium* spp. (0.14%), the outgrowing mycelia were assigned to 23 morphologically different species (Table 2, Figure 2).

All filamentous species found in the isolations were assigned to the Ascomycota. Isolates contained members of the classes Sordariomycetes (nine species, 39.1% of the 23 identified species, 10.2% of all outgrowing fungi), Dothideomycetes (seven, 30.4%, 77.6%), Leotiomycetes (four, 17.4%, 0.5%), Pezizomycetes (three, 13.0%, 1.9%).

The most abundant species was *Sphaeropsis sapinea* (847 isolates, 59.4% of total outgrowing fungi), followed by *Sydowia polyspora* (264, 18.5%), *Truncatella conorum-piceae* (112, 7.9%), *Microsphaeropsis olivacea* (99, 7%), and *Desmazierella acicola* (99, 1.5%). All other species were isolated at a frequency of less than 1%: *Alternaria alternata*, *Biscogniauxia mediterranea*, *Biscogniauxia nummularia*, *Botrytis cinerea*, *Diaporthe* sp., *Epicoccum nigrum*, *Hypoxylon fragiforme*, *Jugulospora rotula*, *Microsphaeropsis olivacea*, *Nemania serpens*, *Pezicula eucrita*, *Pezizomycetes* sp., *Phacidium lacerum*, *Preussia funiculate*, *Pseudocamarosporium brabeji*, *Pyronema domesticum*, *Rosellinia* sp., *Sordaria fimicola*, and *Therrya fuckelii*.

**Figure 3:**
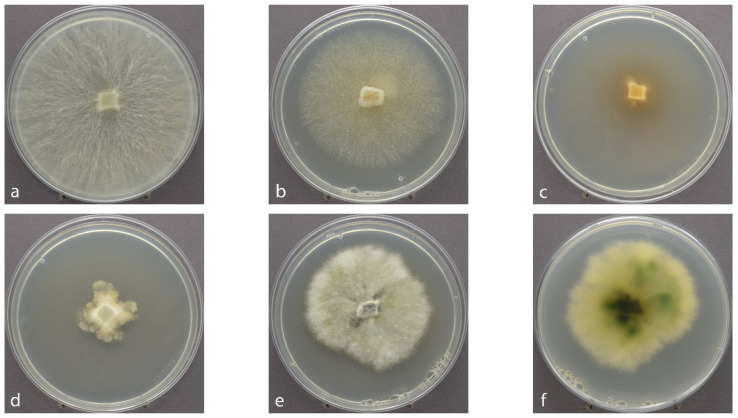
Endophytic Xylariales of Scots pine twigs not detected by HTS, cultivated on MYP in 90 mm Petri dishes, seven days in ambient daylight at room temperature, at around 22 °C: (**a**–**e**) obverse, (**f**) reverse; (**a**) *Biscogniauxia mediterranea*; (**b**) *Biscogniauxia nummularia*; (**c**) *Nemania serpens*; (**d**) *Rosellinia* sp.; (**e**–**f**) *Hypoxylon fragiforme*.

*Ph. lacerum* was only isolated in June, whereas *E. nigrum*, *B. mediterranea*, *Bo. cinerea*, *H. fragiforme*, *N. serpens*, *Py. domesticum*, and *Ps. brabeji* were only isolated in September. More than half of the 23 identified species (65%, Figure 3) were also detected in the HTS data. These included *A. alternata*, *Bo. cinerea*, *E. nigrum*, *Th. fuckelii*, *M. olivacea*, *N. serpens*, *P. eucrita*, *Ph. lacerum*, *Ps. brabeji*, *S. sapinea*, *Sy. polyspora*, and *T. conorum-piceae* (Table S1). The manual categorization into trophic groups based on the authors’ expertise and literature assigned the isolated endophytes as follows:

- 26% pathogenic on conifers (*T. conorum-piceae*, *Bo. cinerea*, *Diaporthe* sp., *Rosellinia* sp., *S. sapinea*, and *Sy. polyspora*).
- 26% typical saprotrophs (*D. acicola*, *Pe. eucrita*, *Ph. lacerum*, *Ps. brabeji*, *Py. domesticum*, and *Th. fuckelii*), except for *Ps. Brabeji*, which is usually found on needles or branches of pine).
- 17% typical hard wood colonizers with lifestyles from endophytic, parasitic to saprotrophs (*B. mediterranea*, *B. nummularia*, *H. fragiforme*, and *N. serpens*).
- 17%, typical generalists with various lifestyles but often saprobic (*A. alternata*, *E. nigrum*, *M. olivacea*, and *S. fimicola*).
- 9% coprophilous species, usually living on soil, dung, or plant debris (*J. rotula* and *Preussia funiculata*).
- The following wood-decay fungi were identified: *B. mediterranea*, *B. nummularia*, and *H. fragiforme*. Except for *Diaporthe* sp., *P. funiculata*, and *Th. fuckelii*, all other isolates of filamentous fungi were identified as typical endophytes of Scots pine twigs in the sense of Bußkamp et al. [20].

**Figure 4:**
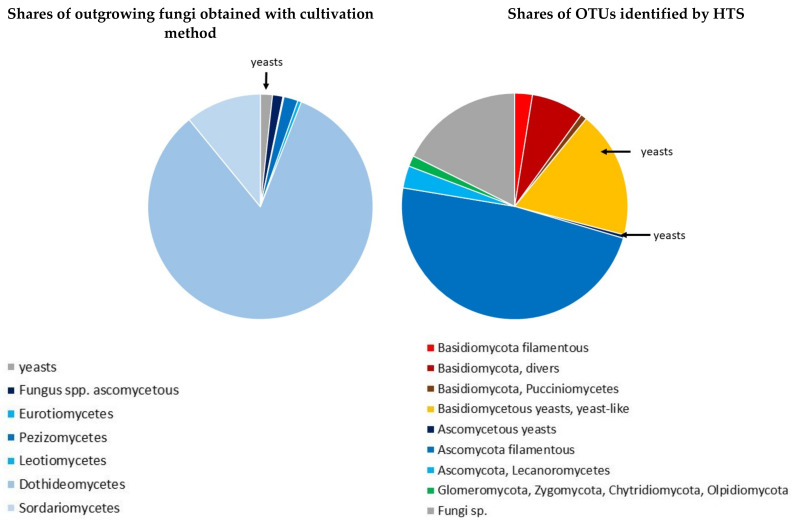
(**left**): Proportions of outgrowing fungi obtained by the cultivation method: (**right**): Proportions of OTUs identified by HTS.

In terms of HTS, a total of 11,684,725 reads was obtained from 95 samples after data cleaning. The average number per sample was 122,997 reads (min 42,537 reads, max 864,376 reads). The reads were assigned to 1233 OTUs (Table S1, Figure 4). Rarefaction curves calculated for each sample (Figure S1) showed that the alpha diversity means varied between samples. The most abundant OTU according to HTS was *Sy. polyspora* (2,537,542 reads, 22%), followed by *S. sapinea* (1,958,770 reads, 17%) and *T. conorum-piceae* (508,355 reads, 4%). *Microsphaeropsis olivacea* abundance was found to be high (197,702 reads, 2%) as well. The variation in reads was high for *S. sapinea* (average 20,619 reads with STDV 111375), followed by *Sy. polyspora* (average 26,710, STDV 56506) and *T. conorum-piceae* (5351 and STDV 5715), highlighting the non-normal distribution of the data.

The observed reads (Figure 4) represented Ascomycota (541 OTUs, 44%), Basidiomycota (311 OTUs, 25%), Chytridiomycota (13 OTUs, 1%), and Glomeromycota (3 OTUs, <1%). In addition, there were OTUs of Olpidiomycota (3 OTUs, <1%) and Zygomycota (1 OTU, <1%), and 367 OTUs (30%) remained unassigned (Table S1). OTUs in Ascomycota could be assigned to Dothideomycetes (195 OTUs, 16%), Eurotiomycetes (69 OTUs, 6%), Leotiomycetes (59 OTUs, 5%), Sordariomycetes (39 OTUs, 3%), Lecanoromycetes (39 OTUs, 3%), Orbiliomycetes (10 OTUs, almost 1%), Incertae sedis (4 OTUs, <1%), Taphrinomycetes (4 OTUs, <1%), Arthoniomycetes (2 OTUs, <1%), Saccharomycetes (2 OTUs, <1%), and Pezizomycetes (1 OTU, <1%). Similarly, in Basidiomycota, OTUs were found representing Tremellomycetes (83 OTUs), Agaricomycetes (49 OTUs, 4%), Cystobasidiomycetes (44 OTUs, almost 4%), Microbotryomycetes (38 OTUs, 3%), Exobasidiomycetes (32 OTUs, almost 3%), Agaricostilbomycetes (23 OTUs, 2%), and Pucciniomycetes (10 OTUs, almost 1%). Wood-decaying fungi, such as OTU1203 (*Vuilleminia* sp.) and OTU1168 (*Stereum* sp.), were also recorded. In Chytridiomycota, all OTUs observed were assigned to Chytridiomycetes (10 OTUs), in Glomeromycota to Glomeromycetes (3 OTUs), and in Zygomycota to Mucoromycetes (1 OTU).

Using the FunGuild script, we were able to assign HTS data for 440 OTUs to thetrophic group. Manual curation led to classification of the identified taxa into endophytes, epiphytes, plant pathogens, and wood-decay fungi (Table S1). Twenty-eight OTUs were possible true endophytes, whilst 20 were epiphytes. Eighty-four OTUs were considered to be plant pathogens, and 12 OTUs were wood-decay fungi.

Most of the OTUs identified by HTS could be manually categorized into trophic groups or lifestyles on the basis of the authors’ expertise and the literature (Figure 4, Table S1). Fifty-nine OTUs were assigned to the black yeasts, including rock-inhabiting fungi or black yeast-like fungi (e.g., OTU7, OTU6, OTU11, and OTU23). Therefore, it is likely that these OTUs had an epiphytic source. Seventeen OTUs were ascomycetous non-black yeasts (e.g., OTU124, OTU555, OTU690: *Taphrina* sp., OTU498: *Taphrina* sp., OTU942: *Debaryomyces* sp.). Some of the latter OTUs may be endophytic, for example, *Debaryomyces* sp., because species of this genus are typical endophytic plant yeasts [89,90]. Other OTUs such as *Taphrina* sp. seem to have an epiphytic source, because they are obligate non-pine host-specific parasites. Five OTUs were identified as ascomycetous, olive-brownish pigmented hyphomycetes with yeast-like growth when young and later producing chlamydospore-like structures (e.g., OTU102: *Neophaeococcomyces catenatus* (de Hoog & Herm.-Nijh.) Crous & M.J. Wingf.). Two hundred and five OTUs were assigned to the basidiomycetous yeasts, yeast-like Basidiomycota, and pleomorphic Basidiomycota with yeast stages including smuts. A big part of this group of OTUs may have an epiphytic source because these taxa are non-pine host-specific parasites such as OTU405, OTU364, OTU411: *Tremella* spp., OTU1263: *Septobasidium* sp., or smuts such as OTU962 and OTU1256. Some of the identified basidiomycetous yeast species (e.g., OTU661, OTU1241, OTU802) belong to the group of typical endophytic yeast genera, such as *Cryptococcus* Vuill. (Tremellales, Agaricomycotina), *Rhodotorula* F.C. Harrison, and *Sporobolomyces* Kluyver & C.B. Niel (both Sporidiobolales, Pucciniomycotina) [89,90]. Twenty-six OTUs were assigned to the Exobasidiaceae, which usually form colonies with single-celled conidia but without hyphae. Members of this basidiomycetous family are commonly non-pine host-specific plant pathogens [91]. In total, 312 (25%) of all OTUs detected with HTS may represent species with yeast or yeast-like stages. Thirty-five OTUs could be classified as filamentous Basidiomycota, including three ectomycorrhizal fungi, whilst 48 basidiomycetous OTUs were impossible to assign to a trophic group or lifestyle. The ectomycorrhizal fungi (OTU177, OTU896, and OTU1088: *Laccaria* spp.) can be assumed to have an epiphytic source as symbiotic, root-associated species. A total of 274 OTUs were assigned to Ascomycota growing with mycelia; this excluded species likely to have an epiphytic source such as lichens or lichenicolous fungi (38 OTUs), fungicolous or obligate non-pine parasitic fungi (6 OTUs), or ascomycetous sooty molds (2 OTUs). The remaining 125 ascomycetous OTUs could not be assigned to a trophic group, as was the case for 372 OTUs, which represented fungi with no significant similarity to sequences in the database. Usually, species of Chytridiomycetes (10 OTUs) inhabit soil, fresh water, or saline estuaries, or are parasitic on, e.g., amphibians. Therefore, it is assumed that chytrid OTUs had an epiphytic source. There were also three Glomeromycota (OTU359, OTU437, and OTU42) that are arbuscular mycorrhizal fungi.

### 3.2. Disease Class

**Figure 5:**
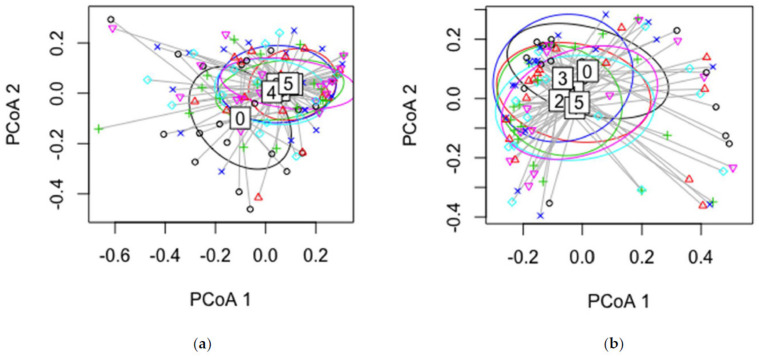
Dispersion of species in the culture-based isolation data (**a**) and OTUs in the HTS data (**b**) for each sample (permutation test using the bray method) in each disease class.

**Figure 6:**
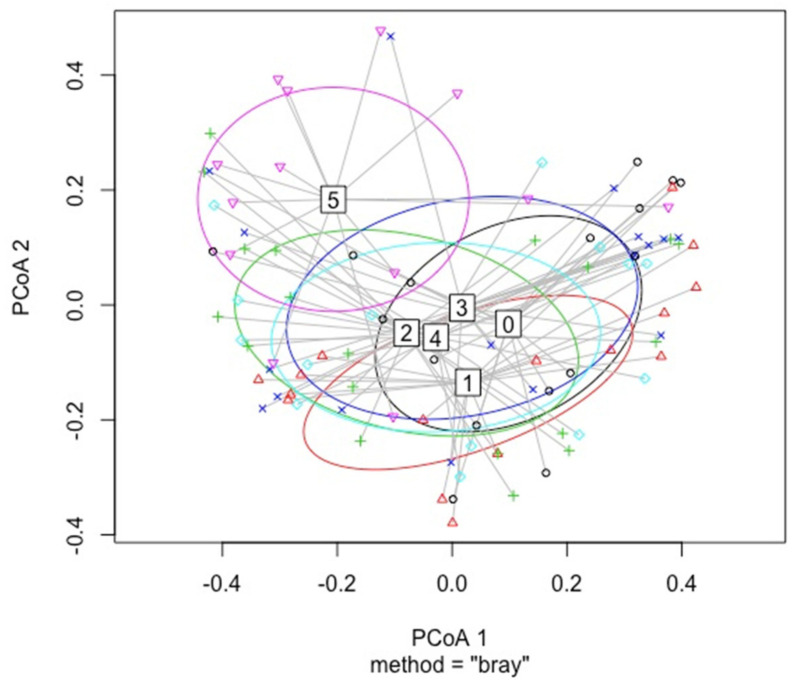
Dispersion of the indicator species of the diseased trees (disease class 5) (67 OTUs) (permutation test using the Bray method) between different disease classes (0–5).

There was no statistical difference between disease classes according to PERMANOVA analysis (*p* = 0.062) (Figure 5a). The diversity indices for the different disease classes were not statistically different in terms of the isolate data, Simpson index (*p* = 0.0625), and Shannon index (*p* = 0.135). On the basis of the “indispecies” analysis, we found that the abundance of the most common endophyte, *M. olivacea*, was statistically different (*p* = 0.0001) between disease class 0 and the other disease classes (according to the isolate data). The HTS data revealed five rare taxa as indicators for disease class 0: *Neophaeothecoidea proteae* (OTU 10), *Botryosphaeriales* sp. 3 IP-2014 (OTU714), *Neodevriesia simplex* (OTU 1192), *Rhizopus oryzae* (OTU1218), and uncultured fungus clone 3980_4 (OTU537). Disease classes from 1 to 4 had 2 to 10 indicator species, and all of them were considered rare (Table S1). Disease class 5 had 67 indicator species (Supplementary Table S1), and an additional PERMANOVA analysis of just these 67 OTUs verified that the different disease classes supported significantly different groups of them (*p* = 0.0032) (Figure 6).

PERMANOVA analysis (*p* = 0.141), the permutation test, and visualization of all HTS data did not reveal any statistical differences in grouping of OTUs (Figure 5b). Similarly, the diversity indices (Shannon *p* = 0.871, Simpson *p* = 0.826) were not statistically different between disease classes. Indispecies and PERMANOVA analyses indicated that overall species diversity in a disease class is similar (abundance and evenness of the species present) between healthy and diseased trees in terms of the HTS data and that changes in communities can be observed only in relation to rare OTUs (Figure 6). The PERMANOVA comparison was also undertaken for HTS reads for the June sampling only (*p* = 0.25) or the September sampling only (*p* = 0.367) and confirmed that there were no statistical differences between disease classes. Similarly, the plant pathogen composition was not statistically different between disease classes (*p* = 0.699). The comparison of these groups confirmed the null hypothesis, in that the centroids and dispersion of the OTUs and diversity indices are equivalent for all groups.

In disease class 0, the defined trophic modes, based on FunGuild results, allowed us to class 55 OTUs as pathotrophs, 98 OTUs as pathotroph-saprotrophs, 114 OTUs as pathotroph-saprotroph-symbiotrophs, 15 OTUs as pathotroph-symbiotrophs, 65 OTUs as saprotrophs, 3 OTUs as saprotrophs-symbiotrophs, and 24 OTUs as symbiotrophs. The averages for each group were not statistically different from each other (Kruskall–Wallis test, *p* = 0.361).

### 3.3. Sampling Time

**Figure 7:**
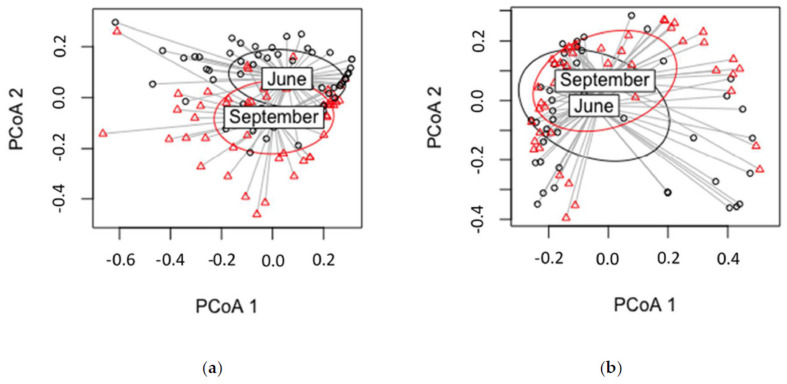
Dispersion of species for the isolate data (**a**) and OTUs in the HTS data; (**b**) in each sample between sampling times June (black) and September (red).

PERMANOVA analysis showed that the species/OTU composition was different between June and September in both isolate (*p* = 0.001) and HTS (*p* = 0.001) data (Figure 7). In isolate data, both Shannon (*p* = 0.00513) and Simpson (*p* = 0.00826) diversity indices were statistically different, indicating higher diversity in September. Diversity indices (Shannon *p* = 0.557, Simpson *p* = 0.225) were not statistically different between sampling times for the HTS data. *Sphaeropsis sapinea* abundance was statistically higher in June in the HTS dataset (*p* = 0.001). *Microsphaeropsis olivacea* (*p* = 0.0001), *T. conorum-piceae* (*p* = 0.0001), and *E. nigrum* (*p* = 0.0486) were statistically more abundant in September in terms of the isolate data. For *Sy. polyspora*, no statistical difference between sampling time was observed.

### 3.4. Year of the Growth

**Figure 8:**
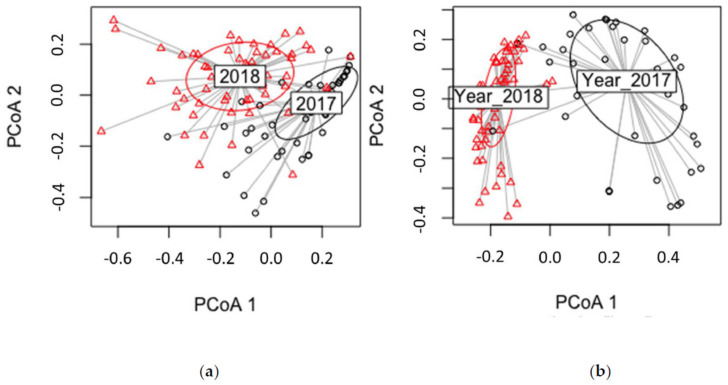
Dispersion of species in the culture-based isolation data (**a**) and OTUs in the HTS data (**b**) between growth years 2017 (black) and 2018 (red).

The species composition in the two years was statistically different according to the data for the culture-based isolated endophytes (*p* = 0.001). Similarly, the composition of OTUs was statistically different (*p* = 0.001) in the HTS data between years of the growth (2017 vs. 2018) (Figure 8). Diversity indices (Shannon, *p* = 3.76 × 10^−5^; Simpson *p* = 5.67 × 10^−7^) for isolate data were significantly different, indicating higher diversity in 2018. Similarly, diversity indices were significantly different for HTS data (Shannon, *p* = 2 × 10^−16^; Simpson’s, *p* = 1.44 × 10^−13^), indicating higher diversity in 2018. On the basis of the HTS data, we found the abundance of *Sy. polyspora* (*p* = 0.0001) and *S. sapinea* (*p* = 0.0008) to be statistically different between growth years. Similarly, in the isolate data the abundance of *Sy. polyspora* (*p* = 0.0001) was statistically different between growth years. In the HTS dataset, *Sy. polyspora* abundance was higher in growth year 2017 (both sampling times), and in the isolate data, it was higher in 2018.

### 3.5. Detection of S. sapinea

**Figure 9:**
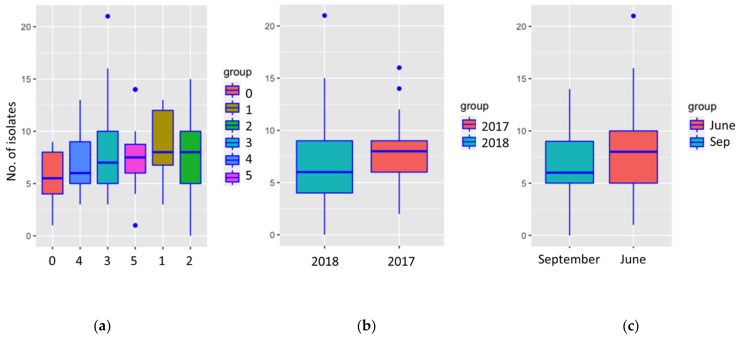
The number of occurrences of *Sphaeropsis sapinea* in different disease classes (**a**), growth years (**b**), and sampling times (**c**) in the culture-based isolation data.

Altogether, 842 (59.44% of all outgrowing fungi) *S. sapinea* strains were isolated from 228 twigs. The number of isolates was not statistically different between disease classes (*p* = 0.0894), time of the year (*p* = 0.459), or time of sampling (*p* = 0.0587) (Figure 9) for the culture-based isolation data.

**Figure 10:**
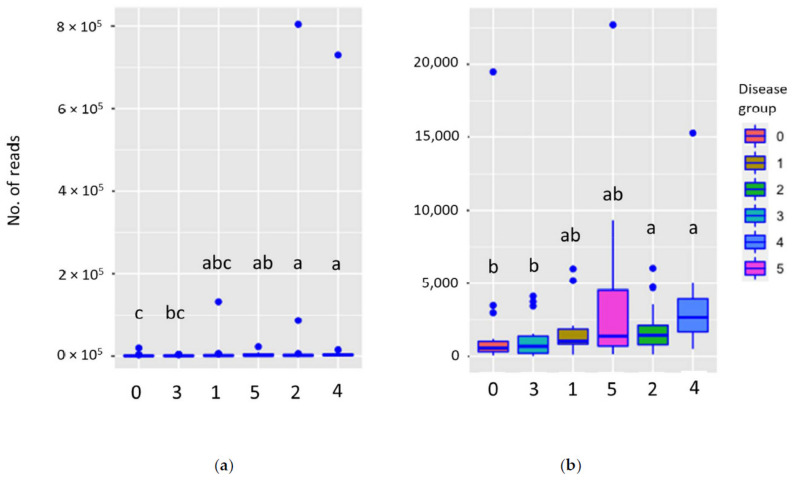
Boxplot of number of *Sphaeropsis sapinea* HTS reads recorded in disease classes (0–5) with (**a**) and without (**b**) outliers.

PERMANOVA analysis of the HTS data showed that *S. sapinea* reads were statistically different between sampling times (*p* = 0.001), growth years (*p* = 0.001), and disease classes (*p* = 0.043). The number of *S. sapinea* reads (HTS data) was higher in June and in growth year 2017. A Kruskall–Wallis test for the HTS data showed that disease class 0 differed significantly from disease classes 2, 4, and 5 (Figure 10a). Similarly, disease class 3 differed from 2 and 4 (Figure 10a). Four outliers, all from June 2017 samples, were detected in the HTS data (disease class 1 = 132,497 reads; disease class 2 = 86,340 and 804,269 reads; disease class 4 = 730,005 reads). After removing these outliers, PERMANOVA analysis showed differences between sampling times (*p* = 0.001), growth years (*p* = 0.012), and disease classes (*p* = 0.019). The numbers of reads were higher in June and in growth year 2017. A Kruskall–Wallis comparison showed that, in disease classes 2 and 4, the number of reads was statistically higher than for disease classes 0 and 3 (Figure 10b). After removing outliers, the average number of reads did not differ statistically between disease classes 0 and 5.

## 4. Discussion

### 4.1. Culture-Based Isolation and HTS Methods Complement Each Other

Selection of methods is crucial for studies aiming to determine the full range of an organism’s microbiome, specifically, in this study, the mycobiome of Scots pine twigs. The large number of OTUs detected with HTS was expected, as large fungal communities have been described before, outstanding in their diversity of morphologies and trophic strategies [56]. The assemblage of cultivable fungi detected only represents the fungi with the ability to metabolize the nutrient medium provided, which grow rapidly enough within the experimental period and are not antagonized by surrounding microbes. HTS, on the other hand, reveals not only endophytes, but also epiphytes and non-cultivable species including yeasts. Nevertheless, the methods complement each other: surprisingly, only 65% of the identified species from the cultivation method were found in the HTS data. Eight species: *B. mediterranea*, *B. nummularia*, *H. fragiforme*, and *Rosellinia* sp. (all Xylariales, Sordariomycetes); *J. rotula* (Sordariales, Sordariomycetes); *D. acicola* and *Py. domesticum* (both Pezizales, Pezizomycetes), and *Pezizomycetes* sp. were not detected by HTS. This indicates that both methods are necessary to reveal the complete mycobiome, especially endophytes. We hypothesized that HTS would be able to detect all fungi, but about half of the species isolated were not recorded. Indeed, the choice of primers and databases still proves to be the bottleneck for the discovery of all species [55,92]. The advantage of the culture-based method is the production of living cultures, which can be tested for virulence, antagonism, ecological relevance, and function.

The main species/OTUs were detected with both methods. Twenty fungi (but not *Diaporthe* sp., *P. funiculata*, and *Th. fuckelii*) isolated in this study were also found in a previous study by Bußkamp et al. [20], in which 103 fungal species were found from 25,800 Scots pine twigs segments (in comparison to 1358 segments in this study). *Th. fuckelii* is a typical endophyte of Scots pine and occurs in the natural distribution area of its host [93]. Usually, it fruits on dead branches, and it is assumed to be member of the fungal self-pruning community of pine [94]. Species of Sordariomycetes (39% of all species isolated by Sanz-Ros et al. [95]) are common endophytic fungi in plant tissues and, as in the current study, Sordariomycetes comprised 31% [20] and 32% [95] of all isolations from Scots pine twigs. *Desmazierella acicola* is a typical saprotroph of Scots pine needles and endophyte of Scots pine twigs [30]. *Py. domesticum* is a pyrophilous cup fungus, occurring on burnt or sterilized soils. It typically fruits within a few weeks of a burn [96].

Endophytic fungi in twigs of *P. sylvestris* identified by culture-based methods have been analyzed by several authors in the past, e.g., [33,95,97,98,99,100]. Regularly isolated species in these and in our studies were *Sy. polyspora*, *M. olivacea*, *S. sapinea*, *D. acicula*, and *Pezicula* spp. As confirmed from studies by Bußkamp et al. [20], some of the isolated endophytes of Scots pine twigs may play a role as decomposers or weak pathogens of the host, e.g., *S. sapinea*, *T. conorum-piceae*, *D. acicola*, *Ph. lacerum*, and *Peniophora pini* (Schleich. ex DC.) Boidin. For most of the isolated endophytes, no significant function is known. However, fungi with ubiquitous or generalist lifestyles commonly isolated from Scots pines, and which probably play a role in the health of the hosts, include *Alternaria*, *Aspergillus*, *Cladosporium*, *Epicoccum*, *Sordaria*, *Phoma*, *Penicillium*, *Phomopsis*, *Pestalotiopsis*, *Xylaria*, and *Nigrospora*. In contrast to the HTS method, basidiomycetous fungi were not found by the culture-based method in this study.

In the current study, the most abundant fungus in all disease classes identified by HTS was the common foliar endophyte of Scots pine, *Sy. polyspora* [33,54]. Similarly, this was the second most common fungus identified by the culture-based method in this study. *Sydowia polyspora* has been found to cause current season needle necrosis (CSNN) in true fir (*Abies* spp.) across Europe and North America [101,102,103], necrosis on shoots of *Pinus pinea* L. [104], and necrosis on stems and needles on *Pinus yunnanensis* Franch. [105]. Cleary et al. [27] suggested that this endophyte is an opportunistic pathogen, in that due to changes in climate, it could increase its pathogenicity. However, Blumenstein et al. [13] showed that drought stress in the host did not increase pathogenicity of *Sy. polyspora*, indicating that it is a true endophyte of Scots pine. Moreover, *Sy. polyspora* has been found to inhibit the growth of *S. sapinea* [13], highlighting its role in host–endophyte interactions.

*Gremmeniella abietina* (Lagerberg) Morelet is a pathogen native to Europe that produces cankers on stems and severe damage, leading to the death of its main host trees, species of *Pinus* and *Picea* [49,106]. Like *S. sapinea*, *G. abietina* causes crown defoliation and distortion of terminal twigs [107], leading to the assumption that it could occupy the same niches in the host tree. We found it from the HTS data but only in five samples (115 reads), indicating that it is not a threat in the study area.

### 4.2. Yeasts—Common Epiphytes of Scots Pine Twigs

The assemblage of fungi detected by the cultivation method only identified a few yeasts (1.61% of outgrowing fungi as endophytes). With the HTS method, 25% of all detected OTUs represented species with yeast or yeast-like stages. There are only few taxa known to be endophytic yeasts and there have been only a few studies of yeasts on conifer species, e.g., *Sequoia sempervirens* (D.Don) Endl [108], *P. sylvestris* [109], and *Pinus tabuliformis* Carrière [110]. Typical endophytic plant yeasts are ascomycetous species of *Debaryomyces* Lodder & Kreger-van Rij (Saccharomycetales, Saccharomycotina), as well as basidiomycetous species of *Cryptococcus* Vuill. (Tremellales, Agaricomycotina), *Rhodotorula* F.C. Harrison, and *Sporobolomyces* Kluyver & C.B. Niel (both Sporidiobolales, Pucciniomycotina) [89,90]. Some of these taxa were found in this study via HTS, e.g., OTU942: *Debaryomyces* sp., OTU499: *Sporobolomyces* sp., OTU1043: *Cryptococcus* sp., or OTU802: *Rhodotorula mucilaginosa*. It is likely that most of the identified yeast species in the current study have an epiphytic source. The number of yeast taxa within shoots, twigs, or stems of trees appears to be low, for example, Middelhoven [108] isolated only four species from young and perennial shoots of *S. sempervirens*: *Debaryomyces hansenii*; *Tausonia pullulans* (Lindner) Xin Zhan Liu; F.Y. Bai, M. Groenew. & Boekhout (≡*Trichosporon pullulans* (Lindner) Diddens & Lodder); and *Trichosporon porosum* (Stautz) Middelhoven, Scorzetti & Fell. Phylloplane yeasts also may influence the behavior, fitness, and growth of their hosts, as they produce plant hormone-like metabolites [53]. Besides filamentous wood decaying fungi, yeasts play an important role during the fungal transformation of wood, e.g., producing a partially de-lignified material. The efficiency in degrading plant material differs between wood-decaying, litter-decomposing, and plant-pathogenic fungi and yeasts [111]. The different decomposer groups differ in their degradation of cellulose and hemicellulose. Yeasts often form an association with basidiomycetes during the wood decay process and are able to consume lignocellulose-related sugars, usually found in tree bark, leaf litter, and rotting wood.

### 4.3. Mycobiome Differences between Sampling Factors

#### 4.3.1. Disease Class

The diversity indices and species/OTU composition did not differ statistically between disease classes, indicating a similar mycobiome in trees of different health classes at the already diseased (Diplodia tip blight) forest site. However, disease class 5 contained most of the indicator species [50], showing that this rare OTU community changes in relation to the health status of the tree. It seems that these few species are benefitting from the decline of the trees. On the basis of our HTS data, we can conclude that there is no measurable change in the core mycobiome between healthy and diseased trees. Due to the fact that core endophytes are the same in diseased and healthy trees, perhaps the ability of the mycobiome to improve host health depends on the strains of fungi. This has been observed several times. For example, Terhonen et al. [112] showed that the ability to inhibit pathogens differs between strains of the same species of root endophytes. Oliva et al. [39]. found that the presence of *Sy. polyspora* was negatively correlated with *S. sapinea* and it is a potential antagonist. This was further supported by Blumenstein et al. [13] as *Sy. polyspora* could inhibit *S. sapinea* in vitro. However, in a study by Bußkamp [33], *Sy. polyspora* did not show any inhibition in dual culture with *S. sapinea*, indicating that the observed antagonism due to fungal endophytes may be strain related. *M. olivacea* has showed antagonisms against *S. sapinea* [13] and it was considered to be indicator species of healthy trees (from isolate data). This highlights the importance of using HTS and isolation methods together when studying possible biocontrol applications.

#### 4.3.2. Sampling Time

As mentioned in several studies [113,114,115], the composition of fungal communities differs over time due to changing weather conditions, the normal cycling of the seasons, and the characteristics of the host plant. In this study, we observed statistical differences between the two sampling times (June versus September), indicating a change in the mycobiome between seasons. In the isolate data, diversity indices revealed higher diversity in September. However, for HTS data, no differences in diversity indices were observed. The amounts of *M. olivacea*, *T. conorum-piceae*, and *E. nigrum* increased in September on the basis of isolate data. Similar observations were made by Martín-Pinto et al. [116], who noted seasonal variation in fungal composition of tree seedlings (including *P. sylvestris*). It seems that the species/OTUs of pine twigs have sophisticated life strategies and species abundance is dependent of the time of the year.

#### 4.3.3. Year of Growth

Compositions of the species/OTUs differed statistically between the growth years. Interestingly, the growth year 2018 had higher diversity indices than 2017 in HTS data. Generally, it is assumed that older tissue has higher diversity and more species [54]. Differences in diversity and frequency of isolations/species/OTUs are correlated with the length of shoots and number of tested segments [20]. However, the shoots from 2017 were often shorter than those from 2018 (Table 1). This increased the statistical difference in favor of 2018. Statistically, there was more *Sy. polyspora* according to the HTS data in the growth year 2017, but the culture-based isolation data revealed that there was more in growth year 2018. It has been observed that the frequency of the fungi increases in older needles [36,54], and we were expecting this to be the case in older twigs. The outcome is clearly related to the size of the segments. As the most common foliar endophyte of Scots pine, *Sy. polyspora* is able to establish itself very well in the current year’s growth and accumulate in the older tissues.

### 4.4. Detection of S. sapinea

*Sphaeropsis sapinea* was the most common fungus of the isolates and the second most common in the HTS analysis. *Sphaeropsis sapinea* has been found in asymptomatic Scots pine trees as an endophyte in several studies [7,20,117]. The disease class of trees (sampled from healthy tissue) as a factor could not explain the accumulation of *S. sapinea* in this study. More multifactorial studies are needed to explain the variables leading to symptom development of Diplodia tip blight. It is likely that the genotype of the tree also has an impact on disease development. Scots pine responds more strongly to pathogen infection than to mycorrhizal or saprotrophic fungi [118]. Mukrimin et al. [119] found that different Scots pine host genotypes exhibited different degrees of susceptibility to *Heterobasidion annosum*. Similar findings have been observed for *Picea abies* as there is genotypic variation in successful spruce defense strategies against *H. annosum* [120]. Screening for potential markers of less/more susceptibility of Scots pine genotypes, as for *H. annosum* infection, should also be applied to *S. sapinea*. Increasing availability of transcriptome data for the Scots pine–*S. sapinea* pathosystem increases opportunities to discover genes that underlie the tree–pathogen interactions along the endophyte–pathogen continuum. This information could ultimately be used to improve future research on tree resistance and create new possibilities in breeding programs.

Besides tree genotype, fungal strains can also have a variable impact on the host and be more or less aggressive [20,121]. The endophytic stage represents a balanced interaction between the fungus and its host. Perhaps the *S. sapinea* strains documented in healthy trees are more adapted to an endophytic lifestyle and are incapable of causing visible disease symptoms [20]. In this study, *S. sapinea* was dominant in culture-based isolation methods, which might indicate that *S. sapinea* is able to grow faster and/or has the ability to outgrow other wood inhabiting fungi in vitro. This hypothetically could also happen in planta. The abundance of *S. sapinea* in the different disease classes, growth years, and sampling times was not found to be different in the isolate data. However, with HTS data, the numbers of reads were different between sampling times, growth years, and disease classes. *S. sapinea* abundance was higher in June and in growth year 2017. *S. sapinea* was more common in June, which coincides with the production of conidial spores of this fungus [122]. As the abundance of *S. sapinea* was higher in older tissues, we conclude that this fungus can, over time, establish a niche in Scots pine woody tissue.

## 5. Conclusions

We conclude that the higher levels of Diplodia tip blight symptoms in this forest stand are not necessarily the result of a higher abundance of *S. sapinea*. Perhaps the health status of these trees is also related to the rare indicator species (67 OTUs). The susceptibility of these trees is probably defined by several abiotic and biotic factors that currently remain unknown. However, in an epidemiological sense, it can be concluded that *S. sapinea* accumulates in the healthy trees without causing symptoms. Indeed, it has previously been reported that the fungus can accumulate for 10 years in a tree before a disease outbreak [25].

We found *S. sapinea* as symptomless endophytic fungi at high abundances in healthy-looking Scots pine trees. This confirms that *S. sapinea* accumulates as an endophyte in healthy trees. Similarly, this observation can explain the sudden and rapid development of disease epidemics in several areas [24,25] and highlight the ability of the fungus to spread unnoticed [7,17,27].

Above, we found no differences in the mycobiome of healthy trees compared to diseased ones. From the core mycobiome, only *M. olivacea* was observed as an indicator species in healthy trees (isolate data). As *M. olivacea* does not cause necrosis of drought-stressed hosts [13] and it can compete with *S. sapinea* in vitro [13], it is one fungus that might be considered for biological applications. The indicator species analysis revealed that, in disease class 5, the rare OTU community is different from that in healthy trees. This change could not have been observed with isolation methods as the community consisted of rare OTUs with unknown functions.

## Figures and Tables

**Table 1:** Year and timing of sample collection for culture-based identification of endophytes and HTS-based identification of the microbiome of Scots pine twigs collected from a total of 35 trees, covering all the disease classes. * Total = the sum of each row.

Sampling Time	June						June						September						September						
Growth year	2017						2018						2017						2018						
Disease class	0	1	2	3	4	5	0	1	2	3	4	5	0	1	2	3	4	5	0	1	2	3	4	5	* Total
No. Scots pine trees sampled in each disease class	5	5	5	5	5	5	5	5	5	5	5	5	5	5	5	5	3	4	5	5	5	5	3	4	35
No. of twigs sampled for HTS	5	5	5	5	5	5	5	5	5	5	5	5	5	5	5	5	5	5	5	5	5	5	5	5	114
Amplicon sequencing successful (HTS)	2	3	5	2	4	1	5	5	5	5	5	5	4	3	4	5	2	4	5	5	5	5	3	3	95
No. of twigs sampled for isolation	10	10	10	10	10	10	10	10	10	10	10	10	10	10	10	10	6	8	10	10	10	10	6	8	228
No. of segments sterilized	47	49	51	50	43	55	56	78	72	90	77	72	54	51	55	40	23	34	58	74	63	69	43	54	1358
No. of isolates	55	48	55	51	42	60	44	71	69	79	66	58	86	60	72	50	38	40	80	71	63	78	46	53	1425
Mean no. of isolates per shoot	5.5	4.8	5.3	5.1	4.2	6	4.4	7.1	6.9	7.9	6.6	5.8	8.6	6.9	7.2	5	4.7	5	8	7.1	6.3	7.8	7.7	6.3	6.83
Mean no. of taxa isolated	3.8	1.8	2.6	2.2	2.8	3.2	5.8	2.4	3.8	2.6	2.7	3	2.2	2.8	3	3.8	2.6	3.2	4.4	4.2	3.8	4.2	5.3	4	-

**Table 2:** Taxa isolated with the culture-based method.

Taxon	Author	Frequency (No. of Isolates/Total No. of Isolations (%))	GenBank Accession Number (This Study)
Yeasts		1.61	not sequenced
Fungus spp. ascomycetous		1.40	not sequenced
*Penicillium* spp.		0.14	not sequenced
*Alternaria alternata*	(Fr.) Keissl.	0.63	MT790311
*Biscogniauxia mediterranea*	(De Not.) Kuntze	0.14	MT790312
*Biscogniauxia nummularia*	(Bull.) Kuntze	0.35	MT790313
*Botrytis cinerea*	Pers.	0.14	MT790314
*Desmazierella acicola*	Lib.	1.54	MT790315
*Diaporthe* sp.		0.91	MT790316
*Epicoccum nigrum*	Link	0.49	MT790317
*Hypoxylon fragiforme*	(Pers.) J. Kickx f.	0.14	MT790318
*Jugulospora rotula*	(Cooke) N. Lundq.	0.07	MT790319
*Microsphaeropsis olivacea*	(Bonord.) Höhn.	6.95	MT790320
*Nemania serpens*	(Pers.) Gray	0.14	MT790321
*Pezicula eucrita*	(P. Karst.) P. Karst.	0.21	MW365343
*Pezizomycetes* sp.		0.14	strain died
*Phacidium lacerum*	Fr.	0.07	not cultivated
*Preussia funiculata*	(Preuss) Fuckel	0.07	MT790322
*Pseudocamarosporium brabeji*	Marinc, M.J. Wingf. & Crous) Crous	0.07	MT790323
*Pyronema domesticum*	(Sowerby) Sacc.	0.21	MT790324
*Rosellinia* sp.		0.21	MT790325
*Sordaria fimicola*	(Roberge ex Desm.) Ces. & De Not.	0.35	not cultivated
*Sphaeropsis sapinea*	(Fr.) Dyko & B. Sutton	59.44	MT790326 andMT790327
*Sydowia polyspora*	(Bref. & Tavel) E. Müll.	18.53	MT790328
*Truncatella conorum-piceae*	(Tubeuf) Steyaert	7.86	MT790329
*Therrya fuckelii*	(Rehm) Kujala	0.07	MT790330

## Data Availability

All metagenome raw data associated with this study have been submitted to the NCBI, SRA database, and can be found using accession number PRJNA645168.

## Supplementary Materials

The following are available online at https://www.mdpi.com/article/10.3390/jof7080607/s1, Figure S1: The alpha diversity mean values as rarefaction curves for each sample of HTS. Table S1: OTU table.
