# Supplementary figures and images for: The Diplodia Tip Blight Pathogen Sphaeropsis sapinea Is the Most Common Fungus in Scots Pines’ Mycobiome, Irrespective of Health Status—A Case Study from Germany
Source: J Fungi (Basel). 2021 Jul 27;7(8):607. doi: 10.3390/jof7080607 (PMC8396920; doi:10.3390/jof7080607)

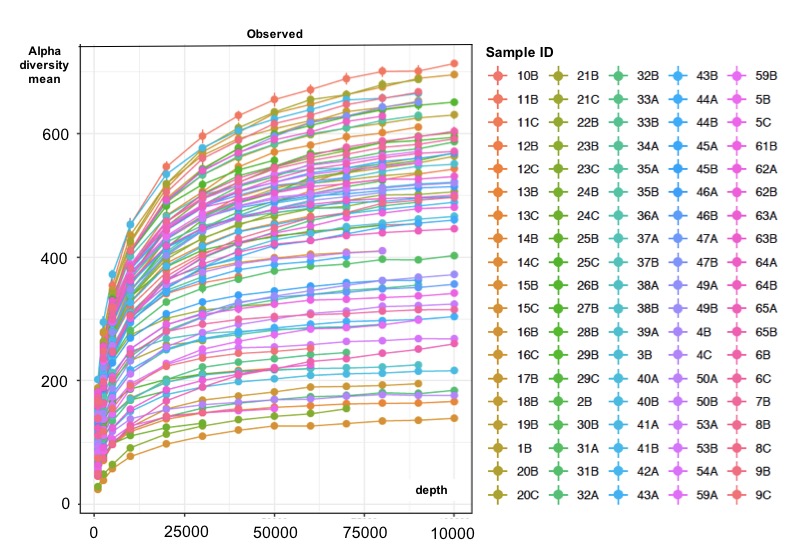

Supplement: Supplementary file 1 [file jof-07-00607-s001.zip › Supplementary Figure 1_14.07.2021.jpg]
